# Supplementary material for: Fungi-based food in the public eye: Terminology, cultivation timelines, sustainability, and nutrition across three EU countries
Source: PLoS One. 2026 Apr 15;21(4):e0345657. doi: 10.1371/journal.pone.0345657 (PMC13082586; doi:10.1371/journal.pone.0345657)
Supplement: S1 File — Includes. (DOCX) [file pone.0345657.s001.docx]

Supplementary materials

**Appendix 1.** Survey questions and statements, Question types, and real-time feedback.

| Master questionnaire | Question | Question type | Correct answer | Real time feedback |
| --- | --- | --- | --- | --- |
|  | Fungi and mushrooms are the same thing and are plants. | true or false | false | Fungi are not plants; they belong to their own kingdom. Mushrooms are the fruiting bodies of certain fungi. Fungi consist of thread-like structures called hyphae, which form a network called mycelium—akin to an underground internet. Mycelium aids plant communication and nutrient sharing, playing a key role in ecosystem health. Fungi have been used in food for over 3,000 years, with examples including Brie cheese, soy sauce, and tempeh |
|  | What is mycoprotein? | multiple choice:  A type of meat substitute;  Proteins in fungi and mushrooms;  A synthetic protein produced in laboratories;  An animal-based protein extracted from marine organisms for enhancing flavor in food;  I do not know | Proteins in fungi and mushrooms | The word mycoprotein describes proteins derived from fungi. |
|  | How long does it take to cultivate filamentous fungi? | multiple choice:  A week or less  Several weeks  Several Months  I do not know | A week or less | Filamentous fungi can grow in as little as one to seven days, depending on the type, environmental control, and nutrition. This growth period is much shorter than that for plants or animals. Cultivation can occur year-round under controlled conditions, independent of seasons. The main benefit is producing high-protein, sustainable, meat-free alternatives that enhance food security and reduce environmental impact |
|  | Cultivating filamentous fungi can play a big role in managing the planet’s resources. | true or false | true | Fungi-based food is a sustainable choice because filamentous fungi can be grown on otherwise wasted food, like unsold bread, and use by-products from other food production, such as oat milk residues. Fungi cultivation requires less water and has a lower carbon footprint and global warming potential compared to plant or animal farming. It also uses less energy |
|  | Fungi-based food can offer a nutritional profile comparable to meat-based products, especially in terms of protein and amino acids. | true or false | true | Fungi-based food can be as nutritious as meat, providing all essential amino acids, vitamins (including E and D2), minerals, antioxidants, and healthy fatty acids. It can contain Omega-3 (ALA), which supports heart health and cholesterol management. Unlike meat, fungi-based food also offers fiber and is considered functional food, providing more than just basic nutrition |
| Swedish questionnaire | Mikrosvampar och storsvampar är samma sak och de är växter. | Sant eller falskt | Falskt | Mikrosvampar är inte växter – de utgör en egen grupp. Storsvampar är en del av vissa mikrosvampar som bildar en fruktkropp. Mikrosvampar består av trådliknande strukturer som kallas hyfer. De bildar nät som kallas mycel och fungerar som ett slags internet under jorden. Mycelet hjälper växterna att kommunicera och dela näring och spelar på så sätt en viktig roll för ekosystemets hälsa. Mikrosvampar har använts i mat i mer än 3 000 år, till exempel i brieost, sojasås och tempeh. |
|  | Vad är mykoprotein? | Multiple choice:  En typ av köttersättningsprodukt;  Proteiner i mikrosvampar och storsvampar;  Ett syntetiskt protein som tillverkas i laboratorier;  Ett animaliskt protein som extraheras från marina organismer för att förhöja matens smak;  Vet inte | Proteiner i mikrosvampar och storsvampar | Ordet mykoprotein beskriver proteiner från mikrosvampar |
|  | Hur lång tid tar det att odla filamentös mikrosvamp? | Multiple choice:  En vecka eller kortare tid;  Flera veckor;  Flera månader;  Jag vet inte | En vecka eller kortare tid | Filamentös mikrosvamp växer snabbt, på 1 till 7 dagar, beroende på typ och näring. De kan odlas året runt i stor skala, oberoende av säsong. Detta skapar förutsättningar för produktion av hållbara, köttfria livsmedelsprodukter med hög proteinhalt, vilket främjar livsmedelstryggheten och minskar miljöpåverkan. |
|  | Odling av filamentös mikrosvamp kan spela en viktig roll när man behöver hushålla med jordens resurser. | Sant eller falskt | Sant | Mikrosvampbaserade livsmedel kan erbjuda stöd i hållbara livsmedelssystem genom att säkra, ätbara resurser som överblivet bröd och biprodukter som havre från tillverkning av havremjölk tas tillvara. Det går åt mindre vatten och energi för att odla filamentös svamp. Dessutom är koldioxidavtrycket samt utsläppen av växthusgaser lägre jämfört med växtodling eller djuruppfödning. Därmed är det ett mer miljövänligt alternativ. |
|  | Mikrosvampbaserade livsmedel kan erbjuda en näringsprofil som är jämförbar med köttbaserade produkter, framför allt vad gäller protein och aminosyror. | Sant eller falskt | Sant | Mikrosvampbaserade livsmedel kan vara lika näringsrika som kött och tillför alla essentiella aminosyror, vitaminer (som E och D2), mineraler, antioxidanter, bioaktiva föreningar, hälsosamma fetter och fibrer. De kan också innehålla omega-3 (ALA), som bidrar till hjärthälsan och att kontrollera kolesterolnivåerna. Till skillnad från kött anses mikrosvamp vara ett funktionellt livsmedel som erbjuder mer än bara basnäring. |
| Spanish questionnaire | Los hongos y las setas son lo mismo y son plantas. | Verdadero o falso | Falso | Los hongos no son plantas; pertenecen a su propio reino. Las setas son los cuerpos fructíferos de ciertos hongos. Los hongos están formados por estructuras filiformes llamadas hifas, que forman una red llamada micelio, similar a una red subterránea de Internet. El micelio ayuda a las plantas a comunicarse y a compartir nutrientes, por lo que juega un papel clave en la salud del ecosistema. Los hongos se utilizan en la alimentación desde hace más de 3000 años, y algunos ejemplos son el queso Brie, la salsa de soja y el tempeh. |
|  | ¿Qué es la micoproteína? | Multiple choice:  Tipo de sustituto de la carne;  Proteínas en hongos y setas;  Una proteína sintética producida en laboratorios;  Una proteína de origen animal extraída de organismos marinos para potenciar el sabor de los alimentos;  No lo sé | Proteínas en hongos y setas | La palabra micoproteína hace referencia a las proteínas derivadas de los hongos. |
|  | ¿Cuánto tiempo se tarda en cultivar hongos filamentosos? | Multiple choice:  Una semana o menos;  Varias semanas;  Varios meses;  No lo sé | Una semana o menos | Los hongos filamentosos crecen rápidamente, entre 1 y 7 días, dependiendo del tipo, el entorno y la nutrición. Pueden cultivarse en grandes extensiones durante todo el año, independientemente de la estación. Esto posibilita la producción de alternativas alimentarias con alto contenido proteínico, sostenibles y sin carne, fomentando la seguridad alimentaria y reduciendo el impacto ambiental. |
|  | El cultivo de hongos filamentosos puede contribuir en gran medida a la gestión de los recursos del planeta. | Verdadero o falso | Verdadero | Los alimentos a base de hongos pueden favorecer sistemas alimentarios sostenibles utilizando recursos seguros y comestibles como el pan duro y derivados, como la avena procedente de la producción de leche de avena. El cultivo de hongos filamentosos precisa menos agua y energía, y tiene una huella de carbono y unas emisiones de gases de efecto invernadero menores que el cultivo de plantas o la cría de animales, lo que lo convierte en una opción más respetuosa con el medio ambiente. |
|  | Los alimentos a base de hongos pueden ofrecer un perfil nutricional comparable al de los productos cárnicos, sobre todo en cuanto a proteínas y aminoácidos. | Verdadero o falso | Verdadero | Los alimentos a base de hongos pueden ser tan nutritivos como la carne, ya que aportan todos los aminoácidos esenciales, vitaminas (como la E y la D2), minerales, antioxidantes, compuestos bioactivos, grasas saludables y fibra. También pueden contener Omega-3 (ALA), que favorece la salud del corazón y el control del colesterol. A diferencia de la carne, los alimentos a base de hongos se consideran funcionales y ofrecen mucho más que una nutrición básica. |
| German questionnaire | Fungi und Pilze sind dasselbe, nämlich Pflanzen. | Richtig oder falsch | Falsch | Fungi sind keine Pflanzen, sondern bilden ein völlig eigenes Reich. Pilze sind die Fruchtkörper einiger bestimmter Fungi. Fungi bestehen aus fadenartigen Strukturen namens Hyphen, die ein Netzwerk bilden, das Myzel, das mit einem Internet im Boden verglichen werden kann. Das Myzel ist für die Kommunikation der Pflanzen und den Nährstoffaustausch wichtig. Es spielt außerdem eine wichtige Rolle für die Gesundheit von Ökosystemen. Fungi werden bereits seit über 3.000 Jahren in Lebensmitteln verwendet, z. B. bei Brie-Käse, Sojasauce und Tempeh. |
|  | Was ist Mykoprotein? | Multipe choice:  Eine Art Fleischersatz;  Protein aus Fungi und Pilzen;  Ein im Labor synthetisch hergestelltes Protein;  Ein tierbasiertes Protein, das aus Meeresorganismen gewonnen und in Lebensmitteln als Geschmacksverstärker eingesetzt wird;  Weiß nicht | Protein aus Fungi und Pilzen | Der Begriff Mykoprotein bezeichnet Protein, das aus Fungi gewonnen wird. |
|  | Wie lange dauert die Zucht von Fadenpilzen? | Multiple choice:  Maximal eine Woche;  Mehrere Wochen;  Mehrere Monate;  Weiß nicht | Maximal eine Woche | Fadenpilze wachsen schnell. Je nach Art, Umgebung und Nährstoffen dauert die Zucht 1 bis 7 Tage. Sie können unabhängig von den Jahreszeiten das ganze Jahr über großflächig angebaut werden. Das ermöglicht die Herstellung von proteinreichen, nachhaltigen, fleischlosen Nahrungsmittelalternativen, die zur Ernährungssicherheit und zum Umweltschutz beitragen. |
|  | Der Anbau von Fadenpilzen kann viel zur Ressourcensicherheit auf unserem Planeten beitragen. | Richtig oder falsch | Richtig | Lebensmittel auf Fungibasis fördern nachhaltige Lebensmittelsysteme, da für sie sichere, essbare Ressourcen wie unverkauftes Brot und Nebenprodukte wie Hafer aus der Hafermilchproduktion genutzt werden. Der Anbau von Fadenpilzen benötigt weniger Wasser und Energie und verursacht einen geringeren CO₂-Fußabdruck sowie weniger Treibhausgasemissionen im Vergleich zum Anbau von Pflanzen oder zur Tierhaltung, was sie zu einer umweltfreundlicheren Option macht. |
|  | Lebensmittel auf Fungibasis können ein ähnliches Nährstoffprofil wie Fleischprodukte bieten, besonders in Bezug auf Protein und Aminosäuren. | Richtig oder faslch | Richtig | Lebensmittel auf Fungibasis können genauso nahrhaft sein wie Fleisch und alle essenziellen Aminosäuren, Vitamine (wie Vitamin E und Vitamin D2), Mineralstoffe, Antioxidantien, bioaktive Stoffe, gesunde Fette und Ballaststoffe enthalten. Zudem können sie Omega-3-Fettsäuren (ALA) enthalten sowie Herzgesundheit und Cholesterinspiegel positiv beeinflussen. Im Gegensatz zu Fleisch gelten fungibasierte Lebensmittel als funktionale Nahrungsmittel, die mehr als nur grundlegende Nährstoffe bieten. |

**Appendix 2.** Sociodemographic profile of the respondents from Germany (DE) n=2002, Spain (ES) n=2002, Sweden (SE) n=2000, and the combined total of respondents n=6004.

| Sociodemographic | Country | Specification | Percentage (%) |
| --- | --- | --- | --- |
| Gender | DE | Female | 51 |
|  |  | Male | 49 |
|  | ES | Female | 52 |
|  |  | Male | 48 |
|  | SE | Female | 50 |
|  |  | Male | 50 |
|  | Combined | Female | 51 |
|  |  | Male | 49 |
| Age | DE | 18-24 years | 9 |
|  |  | 25-34 years | 15 |
|  |  | 35-44 years | 15 |
|  |  | 44-54 years | 18 |
|  |  | 55^+^-years | 43 |
|  | ES | 18-24 years | 8 |
|  |  | 25-34 years | 14 |
|  |  | 35-44 years | 19 |
|  |  | 44-54 years | 19 |
|  |  | 55^+^-years | 39 |
|  | SE | 18-24 years | 5 |
|  |  | 25-34 years | 22 |
|  |  | 35-44 years | 16 |
|  |  | 44-54 years | 16 |
|  |  | 55^+^-years | 40 |
|  | Combined | 18-24 years | 8 |
|  |  | 25-34 years | 17 |
|  |  | 35-44 years | 17 |
|  |  | 44-54 years | 18 |
|  |  | 55^+^-years | 41 |
| Region | DE | Baden-Wuerttemberg | 14 |
|  |  | Bavaria | 16 |
|  |  | Berlin | 4 |
|  |  | Brandenburg | 3 |
|  |  | Bremen | 1 |
|  |  | Hamburg | 4 |
|  |  | Hesse | 9 |
|  |  | Mecklenburg–Western Pomerania | 2 |
|  |  | Lower Saxony | 7 |
|  |  | North Rhine-Westphalia | 22 |
|  |  | Rhineland Palatine | 4 |
|  |  | Saarland | 1 |
|  |  | Saxony | 5 |
|  |  | Saxony Anhalt | 2 |
|  |  | Schleswig Holstein | 3 |
|  |  | Thuringia | 2 |
|  | ES | A1 North | 21 |
|  |  | A2 East | 14 |
|  |  | A3 South | 24 |
|  |  | A4 Center | 22 |
|  |  | A5 Northwest | 9 |
|  |  | A6 North | 9 |
|  | SE | Stockholm County | 23 |
|  |  | Uppsala County | 3 |
|  |  | Södermanland County | 3 |
|  |  | Östergötland County | 5 |
|  |  | Jönköping Country | 4 |
|  |  | Kronoberg County | 1 |
|  |  | Kalmar County | 3 |
|  |  | Gotland County | <1 |
|  |  | Blekinge County | 2 |
|  |  | Scania County | 13 |
|  |  | Halland County | 3 |
|  |  | Västra Götaland County | 16 |
|  |  | Värmland County | 3 |
|  |  | Örebro County | 3 |
|  |  | Västmanland County | 3 |
|  |  | Dalarna County | 3 |
|  |  | Gävleborg County | 3 |
|  |  | Västernorrland County | 3 |
|  |  | Jämtland County | 2 |
|  |  | Västerbotten County | 2 |
|  |  | Norbotten County | 2 |
| Education | DE | Primary | 21 |
|  |  | Upper and post-secondary | 53 |
|  |  | Tertiary | 27 |
|  | ES | Primary | 41 |
|  |  | Upper and post-secondary | 24 |
|  |  | Tertiary | 34 |
|  | SE | Primary | 21 |
|  |  | Upper and post-secondary | 40 |
|  |  | Tertiary | 39 |
|  | Combined | Primary | 28 |
|  |  | Upper and post-secondary | 39 |
|  |  | Tertiary | 33 |
| Household income | DE | Lower income: less than 75% of the median | 42 |
|  |  | Middle income: between 75% and 200% of the median | 39 |
|  |  | Higher income: higher than 200% of the median | 3 |
|  |  | Prefer not to say/Don't know | 16 |
|  | ES | Lower income: less than 75% of the median | 34 |
|  |  | Middle income: between 75% and 200% of the median | 42 |
|  |  | Higher income: higher than 200% of the median | 5 |
|  |  | Prefer not to say/Don't know | 19 |
|  | SE | Lower income: less than 75% of the median | 12 |
|  |  | Middle income: between 75% and 200% of the median | 32 |
|  |  | Higher income: higher than 200% of the median | 3 |
|  |  | Prefer not to say/Don't know | 17 |
|  | combined | Lower income: less than 75% of the median | 29 |
|  |  | Middle income: between 75% and 200% of the median | 38 |
|  |  | Higher income: higher than 200% of the median | 16 |
|  |  | Prefer not to say/Don't know | 17 |
| Eating habits | DE | Vegan (do not eat dairy products, eggs, or any other animal product) | 4 |
|  |  | Vegetarian (do not eat any meat, poultry, game, fish or shellfish) | 6 |
|  |  | Pescatarian (eat fish but do not eat meat or poultry) | 4 |
|  |  | Meat eater (eat meat and/or poultry) | 52 |
|  |  | Flexitarian (Follow a diet of vegetarian-only days, and then mixed diet the rest of the time) | 29 |
|  |  | Other | 4 |
|  |  | Don’t know | 1 |
|  | ES | Vegan (do not eat dairy products, eggs, or any other animal product) | 1 |
|  |  | Vegetarian (do not eat any meat, poultry, game, fish or shellfish) | 2 |
|  |  | Pescatarian (eat fish but do not eat meat or poultry) | 2 |
|  |  | Meat eater (eat meat and/or poultry) | 80 |
|  |  | Flexitarian (Follow a diet of vegetarian-only days, and then mixed diet the rest of the time) | 4 |
|  |  | Other | 8 |
|  |  | Don’t know | 3 |
|  | SE | Vegan (do not eat dairy products, eggs, or any other animal product) | 2 |
|  |  | Vegetarian (do not eat any meat, poultry, game, fish or shellfish) | 4 |
|  |  | Pescatarian (eat fish but do not eat meat or poultry) | 3 |
|  |  | Meat eater (eat meat and/or poultry) | 71 |
|  |  | Flexitarian (Follow a diet of vegetarian-only days, and then mixed diet the rest of the time) | 15 |
|  |  | Other | 3 |
|  |  | Don’t know | 2 |
|  | combined | Vegan (do not eat dairy products, eggs, or any other animal product) | 3 |
|  |  | Vegetarian (do not eat any meat, poultry, game, fish or shellfish) | 4 |
|  |  | Pescatarian (eat fish but do not eat meat or poultry) | 3 |
|  |  | Meat eater (eat meat and/or poultry) | 67 |
|  |  | Flexitarian (Follow a diet of vegetarian-only days, and then mixed diet the rest of the time) | 16 |
|  |  | Other | 5 |
|  |  | Don’t know | 2 |
| Ethnic origin | DE | White | 28 |
|  |  | Black | 0 |
|  |  | South Asian | 1 |
|  |  | East Asian | 1 |
|  |  | Arabic, Central Asian | 1 |
|  |  | Original population (e.g., Inuit, Sami) | 0 |
|  |  | Mixed/dual ethnicity | 1 |
|  |  | Other | 0 |
|  |  | Don’t know | 1 |
|  |  | Do not wish to disclose | 1 |
|  | ES | White | 30 |
|  |  | Black | 0 |
|  |  | South Asian | 0 |
|  |  | East Asian | 0 |
|  |  | Arabic, Central Asian | 0 |
|  |  | Original population (e.g., Inuit, Sami) | 0 |
|  |  | Mixed/dual ethnicity | 1 |
|  |  | Other | 0 |
|  |  | Don’t know | 1 |
|  |  | Do not wish to disclose | 1 |
|  | SE | White | 29 |
|  |  | Black | 0 |
|  |  | South Asian | 0 |
|  |  | East Asian | 0 |
|  |  | Arabic, Central Asian | 1 |
|  |  | Original population (e.g., Inuit, Sami) | 0 |
|  |  | Mixed/dual ethnicity | 1 |
|  |  | Other | 1 |
|  |  | Don’t know | 1 |
|  |  | Do not wish to disclose | 0 |
|  | combined | White | 87 |
|  |  | Black | <1 |
|  |  | South Asian | 1 |
|  |  | East Asian | 1 |
|  |  | Arabic, Central Asian | 2 |
|  |  | Original population (e.g., Inuit, Sami) | <1 |
|  |  | Mixed/dual ethnicity | 2 |
|  |  | Other | 1 |
|  |  | Don’t know | 2 |
|  |  | Do not wish to disclose | 2 |

Appendix 3- Statistical analyses

Subgroup comparisons were conducted using contingency-table analyses. Effect sizes are reported as Cramer’s V for associations between categorical variables. For selected binary outcomes, odds ratios (ORs) with 95% confidence intervals were estimated using logistic regression models, with subgroup reference categories defined as the lowest coded level. Where zero cell counts occurred, a continuity correction was applied. To address multiple comparisons, p-values were adjusted using the Benjamini–Hochberg False Discovery Rate (FDR) procedure within families of related tests (i.e., outcomes within the same subgroup and country). Both unadjusted and FDR-adjusted p-values are reported.

## **Table S1.** Subgroup differences in correct classification of fungi (taxonomy)

| Country | Subgroup variable | Comparison | % Correct | Cramer's V | p | p_FDR (BH) | q < 0.05 |
| --- | --- | --- | --- | --- | --- | --- | --- |
| DE | Gender | Overall (all levels) | 36.8–42.9 | 0.06087 | 0.006458 | 0.0138 | Yes |
| DE | Age | Overall (all levels) | 35.2–54.5 | 0.1131 | 3.805e-05 | 0.0003044 | Yes |
| DE | Region | Overall (all levels) | 35.0–48.5 | 0.08122 | 0.06721 | 0.1075 | No |
| DE | Bundesland | Overall (all levels) | 30.4–58.1 | 0.131 | 0.003033 | 0.01213 | Yes |
| DE | Education - grouped | Overall (all levels) | 34.4–44.5 | 0.0705 | 0.006902 | 0.0138 | Yes |
| DE | Household income – h/m/l | Overall (all levels) | 38.2–43.4 | 0.03049 | 0.6019 | 0.6019 | No |
| DE | Eating habits description | Overall (all levels) | 28.6–46.9 | 0.07149 | 0.1152 | 0.1317 | No |
| DE | Even if you do not identify with these groups/terms, please answer the following for the sake of the survey: How would you best describe your ethnic origin? | Overall (all levels) | 14.7–44.5 | 0.082 | 0.1141 | 0.1317 | No |
| ES | Gender | Overall (all levels) | 61.6–63.0 | 0.01361 | 0.5425 | 0.6329 | No |
| ES | Age | Overall (all levels) | 57.2–76.8 | 0.111 | 5.902e-05 | 0.0002066 | Yes |
| ES | Region | Overall (all levels) | 58.8–65.7 | 0.03779 | 0.7216 | 0.7216 | No |
| ES | Education - grouped | Overall (all levels) | 56.0–71.3 | 0.1387 | 4.364e-09 | 3.055e-08 | Yes |
| ES | Household income – h/m/l | Overall (all levels) | 57.5–68.2 | 0.07496 | 0.01045 | 0.01828 | Yes |
| ES | Eating habits description | Overall (all levels) | 43.0–64.0 | 0.0854 | 0.02359 | 0.03303 | Yes |
| ES | Even if you do not identify with these groups/terms, please answer the following for the sake of the survey: How would you best describe your ethnic origin? | Overall (all levels) | 31.0–66.5 | 0.101 | 0.01033 | 0.01828 | Yes |
| SE | Gender | Overall (all levels) | 64.2–66.8 | 0.02588 | 0.2471 | 0.2825 | No |
| SE | Age | Overall (all levels) | 49.8–68.8 | 0.0886 | 0.003448 | 0.01379 | Yes |
| SE | Region | Overall (all levels) | 59.2–72.2 | 0.07704 | 0.01835 | 0.04012 | Yes |
| SE | Län | Overall (all levels) | 53.0–77.8 | 0.1154 | 0.1455 | 0.2327 | No |
| SE | Education - grouped | Overall (all levels) | 63.1–66.4 | 0.02692 | 0.4845 | 0.4845 | No |
| SE | Household income – h/m/l | Overall (all levels) | 62.4–67.8 | 0.04859 | 0.1933 | 0.2577 | No |
| SE | Which, if any, of these best describes your usual eating habits? | Overall (all levels) | 48.0–72.4 | 0.08668 | 0.02006 | 0.04012 | Yes |
| SE | Even if you do not identify with these groups/terms, please answer the following for the sake of the survey: How would you best describe your ethnic origin? | Overall (all levels) | 33.3–74.6 | 0.1168 | 0.0007244 | 0.005795 | Yes |

## **Table S2**. Subgroup differences in correct identification of mycoprotein (Cramer's V)

| Country | Subgroup variable | Comparison | % Correct | Cramer's V | p | p_FDR (BH) | q < 0.05 |
| --- | --- | --- | --- | --- | --- | --- | --- |
| DE | Gender | Overall (all levels) | 32.4–34.2 | 0.01814 | 0.4169 | 0.5559 | No |
| DE | Age | Overall (all levels) | 29.2–39.2 | 0.07994 | 0.01233 | 0.01973 | Yes |
| DE | Region | Overall (all levels) | 30.5–36.3 | 0.04016 | 0.8631 | 0.9129 | No |
| DE | Bundesland | Overall (all levels) | 28.1–40.8 | 0.06423 | 0.9129 | 0.9129 | No |
| DE | Education - grouped | Overall (all levels) | 22.8–43.3 | 0.1504 | 1.473e-10 | 1.178e-09 | Yes |
| DE | Household income – h/m/l | Overall (all levels) | 30.1–52.2 | 0.08168 | 0.003925 | 0.007851 | Yes |
| DE | Eating habits description | Overall (all levels) | 10.7–39.7 | 0.1247 | 2.385e-05 | 9.541e-05 | Yes |
| DE | Even if you do not identify with these groups/terms, please answer the following for the sake of the survey: How would you best describe your ethnic origin? | Overall (all levels) | 13.4–40.8 | 0.1179 | 0.0007798 | 0.002079 | Yes |
| ES | Gender | Overall (all levels) | 31.2–32.6 | 0.01287 | 0.5647 | 0.6589 | No |
| ES | Age | Overall (all levels) | 23.8–35.7 | 0.06417 | 0.08304 | 0.1453 | No |
| ES | Region | Overall (all levels) | 29.4–38.7 | 0.0537 | 0.3289 | 0.4605 | No |
| ES | Education - grouped | Overall (all levels) | 26.0–36.9 | 0.108 | 8.442e-06 | 5.909e-05 | Yes |
| ES | Household income – h/m/l | Overall (all levels) | 28.8–48.0 | 0.09046 | 0.0009468 | 0.002209 | Yes |
| ES | Eating habits description | Overall (all levels) | 8.5–38.4 | 0.1119 | 0.0003292 | 0.001152 | Yes |
| ES | Even if you do not identify with these groups/terms, please answer the following for the sake of the survey: How would you best describe your ethnic origin? | Overall (all levels) | 15.2–41.4 | 0.04889 | 0.7902 | 0.7902 | No |
| SE | Gender | Overall (all levels) | 32.8–41.7 | 0.09142 | 4.342e-05 | 0.0001458 | Yes |
| SE | Age | Overall (all levels) | 28.5–42.1 | 0.07629 | 0.02024 | 0.02699 | Yes |
| SE | Region | Overall (all levels) | 32.8–40.8 | 0.05328 | 0.2246 | 0.2264 | No |
| SE | Län | Overall (all levels) | 18.7–47.5 | 0.1104 | 0.2264 | 0.2264 | No |
| SE | Education - grouped | Overall (all levels) | 20.8–48.7 | 0.2166 | 4.296e-21 | 3.436e-20 | Yes |
| SE | Household income – h/m/l | Overall (all levels) | 32.3–41.7 | 0.0802 | 0.004942 | 0.007907 | Yes |
| SE | Which, if any, of these best describes your usual eating habits? | Overall (all levels) | 12.8–60.8 | 0.1209 | 5.468e-05 | 0.0001458 | Yes |
| SE | Even if you do not identify with these groups/terms, please answer the following for the sake of the survey: How would you best describe your ethnic origin? | Overall (all levels) | 10.4–50.4 | 0.106 | 0.004637 | 0.007907 | Yes |

## **Table S3**. Odds ratios for correct identification of mycoprotein

| Country | Subgroup | Reference group | Odds ratio | 95% CI (lower) | 95% CI (upper) | p | p_FDR (BH) | q < 0.05 |
| --- | --- | --- | --- | --- | --- | --- | --- | --- |
| DE | Education - grouped: Upper and post secondary | Primary | 1.61 | 1.238 | 2.1 | 0.0003984 | 0.00159 | Yes |
| DE | Education - grouped: Tertiary | Primary | 2.59 | 1.944 | 3.449 | 7.922e-11 | 6.34e-10 | Yes |
| DE | Eating habits description: Vegetarian (do not eat any meat, poultry, game, fish or shellfish) | Vegan (do not eat dairy products, eggs, or any other animal product) | 1.04 | 0.5691 | 1.907 | 0.8946 | 0.91 | No |
| DE | Eating habits description: Pescatarian (eat fish but do not eat meat or poultry) | Vegan (do not eat dairy products, eggs, or any other animal product) | 0.962 | 0.4926 | 1.879 | 0.9098 | 0.91 | No |
| DE | Eating habits description: Meat eater (eat meat and/or poultry) | Vegan (do not eat dairy products, eggs, or any other animal product) | 0.941 | 0.5828 | 1.521 | 0.8054 | 0.91 | No |
| DE | Eating habits description: Flexitarian (Follow a diet of vegetarian-only days, and then mixed diet the rest of the time) | Vegan (do not eat dairy products, eggs, or any other animal product) | 1.34 | 0.8182 | 2.181 | 0.2469 | 0.395 | No |
| DE | Eating habits description: Other | Vegan (do not eat dairy products, eggs, or any other animal product) | 0.349 | 0.1622 | 0.7507 | 0.007066 | 0.0188 | Yes |
| DE | Eating habits description: Don't know | Vegan (do not eat dairy products, eggs, or any other animal product) | 0.244 | 0.06643 | 0.8952 | 0.03343 | 0.0669 | No |
| ES | Education - grouped: Upper and post secondary | Primary | 1.53 | 1.202 | 1.955 | 0.000567 | 0.00227 | Yes |
| ES | Education - grouped: Tertiary | Primary | 1.66 | 1.337 | 2.073 | 5.161e-06 | 4.13e-05 | Yes |
| ES | Eating habits description: Vegetarian (do not eat any meat, poultry, game, fish or shellfish) | Vegan (do not eat dairy products, eggs, or any other animal product) | 2.6 | 0.8085 | 8.353 | 0.1089 | 0.216 | No |
| ES | Eating habits description: Pescatarian (eat fish but do not eat meat or poultry) | Vegan (do not eat dairy products, eggs, or any other animal product) | 2.55 | 0.7649 | 8.477 | 0.1277 | 0.216 | No |
| ES | Eating habits description: Meat eater (eat meat and/or poultry) | Vegan (do not eat dairy products, eggs, or any other animal product) | 2.1 | 0.7668 | 5.74 | 0.149 | 0.216 | No |
| ES | Eating habits description: Flexitarian (Follow a diet of vegetarian-only days, and then mixed diet the rest of the time) | Vegan (do not eat dairy products, eggs, or any other animal product) | 2.09 | 0.6963 | 6.265 | 0.1888 | 0.216 | No |
| ES | Eating habits description: Other | Vegan (do not eat dairy products, eggs, or any other animal product) | 1.2 | 0.4128 | 3.475 | 0.7398 | 0.74 | No |
| ES | Eating habits description: Don't know | Vegan (do not eat dairy products, eggs, or any other animal product) | 0.39 | 0.09611 | 1.582 | 0.1875 | 0.216 | No |
| SE | Education - grouped: Upper and post secondary | Primary | 2.02 | 1.534 | 2.672 | 6.42e-07 | 6.42e-07 | Yes |
| SE | Education - grouped: Tertiary | Primary | 3.61 | 2.741 | 4.753 | 0 | 0 | Yes |

## **Table S4**. Subgroup differences in awareness of filamentous fungi cultivation time

| Country | Subgroup variable | Comparison | % Correct | Cramer's V | p | p_FDR (BH) | q < 0.05 |
| --- | --- | --- | --- | --- | --- | --- | --- |
| DE | Gender | Overall (all levels) | 18.9–22.4 | 0.04154 | 0.06305 | 0.1009 | No |
| DE | Age | Overall (all levels) | 15.3–30.2 | 0.155 | 9.1e-10 | 3.64e-09 | Yes |
| DE | Region | Overall (all levels) | 17.8–29.2 | 0.06638 | 0.2657 | 0.3543 | No |
| DE | Bundesland | Overall (all levels) | 13.1–27.4 | 0.08075 | 0.5981 | 0.5981 | No |
| DE | Education - grouped | Overall (all levels) | 16.0–29.8 | 0.1391 | 3.834e-09 | 1.022e-08 | Yes |
| DE | Household income – h/m/l | Overall (all levels) | 16.9–36.4 | 0.1098 | 2.32e-05 | 4.641e-05 | Yes |
| DE | Eating habits description | Overall (all levels) | 10.5–61.2 | 0.2249 | 1.349e-19 | 1.079e-18 | Yes |
| DE | Even if you do not identify with these groups/terms, please answer the following for the sake of the survey: How would you best describe your ethnic origin? | Overall (all levels) | 7.4–29.8 | 0.06572 | 0.4041 | 0.4618 | No |
| ES | Gender | Overall (all levels) | 17.6–21.9 | 0.0538 | 0.01608 | 0.06295 | No |
| ES | Age | Overall (all levels) | 16.1–22.6 | 0.06145 | 0.1091 | 0.2547 | No |
| ES | Region | Overall (all levels) | 16.7–23.7 | 0.0511 | 0.3888 | 0.5326 | No |
| ES | Education - grouped | Overall (all levels) | 18.5–21.3 | 0.02799 | 0.4565 | 0.5326 | No |
| ES | Household income – h/m/l | Overall (all levels) | 18.1–20.6 | 0.02179 | 0.8131 | 0.8131 | No |
| ES | Eating habits description | Overall (all levels) | 6.6–33.7 | 0.08745 | 0.01798 | 0.06295 | No |
| ES | Even if you do not identify with these groups/terms, please answer the following for the sake of the survey: How would you best describe your ethnic origin? | Overall (all levels) | 11.4–37.8 | 0.05856 | 0.2479 | 0.4339 | No |
| SE | Gender | Overall (all levels) | 20.6–29.9 | 0.1054 | 2.423e-06 | 9.691e-06 | Yes |
| SE | Age | Overall (all levels) | 23.9–28.8 | 0.04229 | 0.4663 | 0.4663 | No |
| SE | Region | Overall (all levels) | 21.1–27.5 | 0.0459 | 0.378 | 0.432 | No |
| SE | Län | Overall (all levels) | 12.2–53.2 | 0.1136 | 0.1723 | 0.2298 | No |
| SE | Education - grouped | Overall (all levels) | 16.4–31.3 | 0.1282 | 7.317e-08 | 5.853e-07 | Yes |
| SE | Household income – h/m/l | Overall (all levels) | 20.2–29.9 | 0.08811 | 0.001418 | 0.00378 | Yes |
| SE | Which, if any, of these best describes your usual eating habits? | Overall (all levels) | 9.0–26.9 | 0.0735 | 0.09464 | 0.1514 | No |
| SE | Even if you do not identify with these groups/terms, please answer the following for the sake of the survey: How would you best describe your ethnic origin? | Overall (all levels) | 3.1–50.9 | 0.09323 | 0.0286 | 0.0572 | No |
