# Supplementary material for: Fungi-based food in the public eye: Terminology, cultivation timelines, sustainability, and nutrition across three EU countries
Source: PLoS One. 2026 Apr 15;21(4):e0345657. doi: 10.1371/journal.pone.0345657 (PMC13082586; doi:10.1371/journal.pone.0345657)
Supplement: Attachment — Submitted filename: Response to Remaining Minor comments.docx [file pone.0345657.s004.docx]

**Response to Remaining Minor Revisions Required**

We appreciate all these comment that have further improve the quality of this manuscript. Here comes responses point by point:

1) Figure/Table Consistency: Ensure all Figures 1–5 and Tables 1–10 are present, correctly numbered, and include captions; replace any placeholders (e.g., “image[[…]]”) with actual figures.

**Response:**

We have ensured that all referenced figures (Figures 1–5) and tables (Tables 1–10) are included in the manuscript file, are correctly numbered, and have accompanying captions. Any placeholder content has been removed and the actual figure images are embedded in the manuscript near the corresponding captions.

*Manuscript change:*

Figures are placed in the Results/Discussion section directly adjacent to their captions (Figures 1–5).

Tables 1–10 appear in sequence in the Results/Discussion with captions immediately above each table.

2) Reference Formatting: Verify reference list completeness and PLOS ONE style; ensure all in-text citations match; remove duplicates.

**Response:**

We reviewed and corrected the reference list to ensure it is complete, consistently formatted, and aligned with PLOS ONE numeric citation conventions. We confirmed that every in-text citation has a corresponding reference-list entry and verified that no duplicate references remain.

*Manuscript change:*

References section: checked for completeness, consistency, and duplicates; standardized formatting (author list, year, title, source, volume/issue/pages, DOI/URL where available).

3) Data Availability Statement + Minimal Dataset: Confirm statement is accurate and that Supporting Information includes the minimal dataset required to replicate findings.

**Response:**

We added/confirmed a Data Availability statement in the manuscript and have ensured that the Supporting Information package is prepared to include the minimal dataset required to replicate the analyses.

4) Ethics Statement: Ensure ethics approval number “2024-04842-01” is consistently reported and matches documentation.

**Response:**

We verified that the ethics approval information is stated consistently and that the approval number is reported as “2024-04842-01”.

*Manuscript change:*

Materials and methods → Panel recruitment includes the ethics approval statement and the approval number.

5) Final Proofread: Final proofread for residual typos/formatting; stray fragments; inconsistent symbols; correct supplementary appendix references.

Response:

We conducted a full proofread and corrected remaining typographical and formatting inconsistencies, checked for stray fragments, and harmonized symbol/terminology usage throughout. We also checked that all Supporting Information/appendix references are consistent with the files prepared for submission.

*Manuscript change:*

Proofread across all sections, with particular attention to Methods, Results tables/captions, end matter, and Supporting Information callouts.
