# Supplementary material for: Fungi-based food in the public eye: Terminology, cultivation timelines, sustainability, and nutrition across three EU countries
Source: PLoS One. 2026 Apr 15;21(4):e0345657. doi: 10.1371/journal.pone.0345657 (PMC13082586; doi:10.1371/journal.pone.0345657)
Supplement: Attachment — Submitted filename: Responses to Reviewer comments.docx [file pone.0345657.s003.docx]

**Journal Requirements:**

When submitting your revision, we need you to address these additional requirements.

1.Please ensure that your manuscript meets PLOS ONE's style requirements, including those for file naming. The PLOS ONE style templates can be found at

<https://journals.plos.org/plosone/s/file?id=wjVg/PLOSOne_formatting_sample_main_body.pdf> and

[https://journals.plos.org/plosone/s/file?id=ba62/PLOSOne_formatting_sample_title_authors_affiliations.pdf](https://track.editorialmanager.com/CL0/https:%2F%2Fjournals.plos.org%2Fplosone%2Fs%2Ffile%3Fid=ba62%2FPLOSOne_formatting_sample_title_authors_affiliations.pdf/1/010f019b0de0bd8b-1aa60e27-55e3-4942-817d-6285b9cd94c4-000000/Zg311rTYWYTMzGRlHCp0Ck9EbNbMbN9I4c4Kphm8YtY=241)

**Response**: Yes, we have followed the style requirement.

2. Please note that funding information should not appear in any section or other areas of your manuscript. We will only publish funding information present in the Funding Statement section of the online submission form. Please remove any funding-related text from the manuscript.

**Response**: We have removed the grant number from the manuscript.

3. Thank you for stating the following financial disclosure:

“Swedish Research Council FORMAS with grant number 2023-02018”

Please state what role the funders took in the study.  If the funders had no role, please state: "The funders had no role in study design, data collection and analysis, decision to publish, or preparation of the manuscript."

If this statement is not correct you must amend it as needed.

Please include this amended Role of Funder statement in your cover letter; we will change the online submission form on your behalf.

**Response**: We have added the above sentence.

4. In the online submission form, you indicated that “All relevant data are within the manuscript and its Supporting Information files. More data will be available upon request.”

All PLOS journals now require all data underlying the findings described in their manuscript to be freely available to other researchers, either 1. In a public repository, 2. Within the manuscript itself, or 3. Uploaded as supplementary information.

This policy applies to all data except where public deposition would breach compliance with the protocol approved by your research ethics board. If your data cannot be made publicly available for ethical or legal reasons (e.g., public availability would compromise patient privacy), please explain your reasons on resubmission and your exemption request will be escalated for approval.

**Response**: We have now all relevant data are within the manuscript and its Supporting Information files.

5. If the reviewer comments include a recommendation to cite specific previously published works, please review and evaluate these publications to determine whether they are relevant and should be cited. There is no requirement to cite these works unless the editor has indicated otherwise.

**Response**: Thanks! We considered this point.

**Additional Editor Comments:**

This manuscript presents a timely and well-executed descriptive study on public knowledge and perceptions of fungi-based foods across three distinct EU countries. The topic is highly relevant to sustainable food systems, consumer science, and science communication. The study's strengths are considerable: a large, professionally collected sample (n=6,004), a clear cross-national comparative design, and a focus on multiple, concrete domains of knowledge (taxonomy, terminology, cultivation, sustainability, nutrition). The findings, particularly the coexistence of favorable general attitudes with significant factual misconceptions, are informative and have clear implications for public communication and market development. However, the manuscript in its current form exhibits significant conceptual, methodological, and interpretive shortcomings that prevent acceptance. It reads more as a detailed report of survey findings than as a rigorous academic contribution that critically engages with theory, measurement validity, and the limits of inference. Substantial revision is required to elevate the work to the standard expected by PLoS ONE.

**Detailed Critical Evaluation:
1. Conceptual and Theoretical Framing**
• Vague Constructs: The core concepts of “public literacy,” “process literacy,” and “knowledge” are used interchangeably without clear definition or theoretical grounding. The study operationalizes “literacy” via five isolated quiz items. This is a severe limitation. The manuscript must explicitly define what constitutes “knowledge” or “literacy” in this context (e.g., declarative factual knowledge, conceptual understanding, functional application) and justify why the selected items are valid proxies for these constructs. A brief theoretical discussion linking to models of science literacy or food technology acceptance (e.g., the Cognitive Mediation Model, Food Technology Neophobia) would provide necessary depth.
• Overstated Novelty: The claim that empirical evidence on public knowledge “remains unstudied" (L52-53) is inaccurate, as the authors' own prior work (Hellwig, 2023; Hellwig et al., 2024) explores related consumer perceptions. The contribution should be reframed more precisely: this study provides the first large-scale, cross-national quantification of specific factual knowledge gaps, complementing existing qualitative and attitudinal research.

**Response**: We appreciate all comments by the respected editor and reviewers. Thank you also for this important point. We agree that our original wording could be read as treating “literacy” and “knowledge” interchangeably, and that the construct boundaries required clearer definition and theoretical justification. We have revised the manuscript to explicitly define the construct we measure as domain-specific factual knowledge (declarative recognition of foundational statements about fungi-based foods) and to distinguish it from broader, skills-based notions of “science literacy.” We further clarify process-related knowledge as a subset of factual knowledge focusing on production/cultivation attributes (e.g., cultivation timelines). To avoid conceptual overreach, we have reduced use of the term “literacy” and present the five items as indicators of specific, communication-relevant knowledge gaps, rather than as a validated psychometric scale.

We also strengthened the theoretical framing by linking knowledge acquisition and food-technology acceptance: we now situate the study within established perspectives where information exposure and cognitive processing support knowledge formation (e.g., the Cognitive Mediation Model) and where familiarity/technology-related reluctance can shape openness to novel food production methods (e.g., Food Technology Neophobia). Finally, we expanded the Limitations section to explicitly acknowledge the restricted scope of five closed-ended items and the distinction between declarative knowledge and deeper conceptual or functional understanding, and we outline how future work should validate multi-item scales and test causal pathways experimentally. We have made these revision in the manuscript:

- Introduction: added construct definitions and a short theoretical framing paragraph; tightened terminology to “factual knowledge”/“process-related knowledge” rather than “literacy.”
- Materials and methods: revised the description of the five knowledge items to clarify what they measure and what they do not measure (not a validated scale).
- Abstract/Results & discussion/Conclusion: replaced broad “literacy” claims with accurate “factual knowledge” language.
- Limitations: added explicit measurement and sampling limitations and recommended next-step research designs.

**2. Methodological Rigor and Transparency**
• Measurement Validity Concerns: This is the most critical flaw.
o Compound Question (Taxonomy): The true/false item “Fungi and mushrooms are the same thing and are plants” combines two distinct propositions. A respondent could correctly know fungi are not plants but believe “mushroom” is a synonym for “fungus,” leading to an incorrect answer. This conflates different types of misunderstanding and compromises the validity of inferences drawn from this item. The authors must acknowledge this limitation prominently and temper conclusions accordingly.
o Ambiguous Terminology: The “mycoprotein” question assumes a single correct definition ("proteins in fungi and mushrooms"), while the text later acknowledges the term's variable use in regulatory and market contexts. This creates a validity issue: is the item testing public understanding of a scientific term or familiarity with a specific industry usage? This ambiguity must be discussed.
o Ceiling Effects & Acquiescence Bias: The extremely high agreement on sustainability (85-88%) and nutrition (82-85%) items suggests possible ceiling effects. The positively framed, declarative statements ("can play a big role," "can offer a comparable profile") are susceptible to acquiescence bias (agreement due to positive phrasing rather than conviction). The interpretation of these results as indicating “broad endorsement” or “soft acceptance” is therefore tenuous. The discussion must explicitly acknowledge that these items likely measure general, untested beliefs or openness to claims, not deep knowledge.
• Sampling and Generalizability: The use of a proprietary, opt-in online panel (YouGov) is adequately described but its limitations are underplayed. The sample is not probabilistic, and digitally engaged panels may systematically differ from the general population in education, income, and interest in novel topics. The manuscript must include a stronger caveat about generalizability, particularly for prevalence estimates. The missing Appendix 2 (sociodemographic profile) is essential for evaluating representativeness and must be provided.
• Statistical Reporting: The reporting is inadequate for a study of this scale and complexity.
o Lack of Effect Sizes: With n=6,004, even trivial differences will be statistically significant. Reporting only p-values without effect sizes (e.g., Cramer's V for proportions, odds ratios from logistic regressions) is misleading and prevents readers from judging the practical significance of the dozens of subgroup differences reported. This must be corrected.
o Multiple Comparisons: The manuscript conducts a vast number of statistical tests across countries and numerous sociodemographic subgroups. The risk of Type I errors (false positives) is extremely high. There is no mention of correction for multiple comparisons (e.g., Bonferroni, False Discovery Rate) nor a caution to interpret subgroup findings with extreme care. This is a major statistical oversight that weakens all subgroup analyses.

**Response**: We appreciate also these comments.

For Measurement Validity Concerns

(a) Compound Question (Taxonomy)

*Response:* We agree this item combines two distinct propositions and therefore cannot isolate which misconception drove an incorrect response. We revised the manuscript to (i) explicitly acknowledge this construct-validity limitation, (ii) interpret the result as a composite taxonomy-related misconception rather than a single diagnostic indicator, and (iii) temper conclusions accordingly. We have made these revision in the manuscript:

- Materials and methods → Data collection: added item-level caution about the compound structure.
- Results 3.1 (taxonomy): added a paragraph stating the item captures a composite misconception and cannot distinguish which proposition respondents misunderstood.
- Limitations: added a prominent limitation statement and tempered inference language.

(b) Ambiguous Terminology (“mycoprotein”)

*Response:* We agree that “mycoprotein” can have variable usage across regulatory and market contexts. We clarified that the survey operationalized the term using a simplified, lay definition suitable for cross-national fielding and that responses should be interpreted as familiarity with a common usage/label rather than knowledge of a single authoritative definition. We have revised:

- Materials and methods → Data collection: clarified interpretive scope of the mycoprotein item.
- Results 3.2: added a paragraph explicitly discussing definitional variability and the resulting validity implication.
- Limitations: added a dedicated limitation sentence for the mycoprotein item.

(c) Ceiling Effects & Acquiescence Bias (sustainability/nutrition items)

*Response:* We agree that the very high agreement levels suggest ceiling effects and that the positively framed statements may be susceptible to acquiescence bias. We revised the Discussion to interpret these items as capturing general beliefs/openness to claims rather than deep knowledge, and we tempered language suggesting “endorsement” or “acceptance”. Here are the revisions:

- Materials and methods → Data collection: added a caution that these items are positively framed and may measure beliefs/openness.
- Results 3.4 and 3.5: added explicit discussion of ceiling effects and acquiescence risk and tightened interpretation language.
- Conclusion: removed/tempered “broad endorsement/soft acceptance” framing.
- Limitations: added an explicit statement that these items likely reflect general beliefs/openness, not tested understanding.

**3. Interpretation and Discussion**
• Overinterpretation of Descriptive Data: The manuscript frequently ventures into speculative, post-hoc explanations for observed patterns without supporting data. Examples include:
o Explaining the German taxonomy result via outdated biology education, a factor presumably similar across Europe.
o Attributing lower accuracy among Swedish vegans to a "plant vs. animal" worldview dichotomy.
o Linking Swedish mycoprotein knowledge to mushroom foraging culture.
While interesting as hypotheses, these are not conclusions supported by the study design. The discussion must clearly demarcate between observed patterns and speculative explanations, labeling the latter as such and suggesting them as avenues for future research.
• Inferential Leaps: The claim that high agreement on sustainability/nutrition statements indicates "soft acceptance" is a significant inferential leap. Acceptance is a multi-faceted construct (affective, cognitive, behavioral) not measured here. The data show only agreement with broad, positive statements. This conclusion should be removed or heavily qualified.
• Circular Argument (Nutrition): The discussion uses evidence from protein synthesis studies to “validate” the high public agreement on the nutrition item. This is somewhat circular, as the item specifically asks about protein/amino acid parity. A more nuanced discussion would acknowledge that while protein quality may be comparable, the total nutritional profile (e.g., iron, B12, cholesterol) is more complex, which could justify a "false" answer from a nutritionally sophisticated respondent.
• Blurring of Public Perception and Scientific Evidence: Sections 3.4 and 3.5 oscillate between reporting survey results and reviewing scientific literature (LCAs, feeding studies). This creates a conflation whereby high public agreement might be misread as validating the scientific claims. The structure should be revised to clearly separate: 1) what the public believes, and 2) what the scientific evidence says.

**Response**: Thank you for this important point. We agree that our original Discussion occasionally moved beyond what can be supported by the survey design. We have revised the Discussion to (i) clearly distinguish observed survey patterns from hypothesized explanations, (ii) avoid framing agreement with positively worded statements as “acceptance,” and (iii) structurally separate the reporting of public beliefs from the summary of relevant scientific evidence.

**Regarding Overinterpretation of descriptive data / post-hoc explanations**

*Response:* We revised the Discussion to explicitly label potential mechanisms as hypotheses and to avoid causal or explanatory claims not measured in our data. Specifically, we edited the discussion of (a) the age gradient in the taxonomy item, (b) dietary subgroup differences (including the Swedish vegan/vegetarian pattern), and (c) cross-national differences in mycoprotein knowledge to clarify that these are speculative interpretations and proposed avenues for future research. We also added an explicit statement early in the Results & Discussion section clarifying that the study is descriptive and does not measure mediators such as curriculum exposure, media use, or product familiarity; therefore, mechanistic interpretations are presented only as hypotheses.

**Regarding Inferential leaps: agreement ≠ acceptance**

*Response:* We agree. We removed/rewrote language implying that high agreement with the sustainability and nutrition statements indicates “acceptance.” The revised manuscript now interprets these responses as agreement with broad, positively framed claims and explicitly notes that acceptance is multi-dimensional (affective/cognitive/behavioral) and was not measured here. We also explicitly acknowledge susceptibility to ceiling/acquiescence effects when interpreting these items.

**Regarding Circular argument regarding nutrition**

*Response:* We revised the nutrition discussion to avoid using scientific feeding/protein studies to “validate” the survey responses. The manuscript now treats the scientific literature as context that may help explain why a claim is perceived as plausible, while emphasizing that the survey measured agreement with a statement rather than nutritional literacy. We also added a more nuanced discussion noting that “nutritional profile” extends beyond amino-acid composition (e.g., micronutrients and other attributes), such that a nutritionally sophisticated respondent could reasonably disagree with a blanket parity statement depending on interpretation.

**Regarding Blurring of public perception and scientific evidence (Sections 3.4 and 3.5)**

*Response:* We restructured Sections 3.4 and 3.5 to clearly separate: (1) survey results (public beliefs) and (2) a clearly labeled “scientific evidence context” subsection, explicitly described as separate from what the survey measured. We further added clarifying language that survey agreement should not be read as validating the magnitude of environmental or nutrition impacts.

**4. Structural and Presentational Issues**
• Missing Supplementary Material: Appendix 2 (sociodemographics) is referenced but absent. The full survey instrument with exact wording and response options for all languages should be included.
• Technical Errors: Several need correction: typo “recruitement” (L76), formatting error "fungiand" (L197), inconsistent grant number (2013 vs 2023), duplicate reference entry for Hellwig et al., lowercase start at L538.
• Conclusion: The conclusion is a list of communication recommendations derived directly from the findings. While practical, it lacks a synthesizing statement on the study's broader theoretical or empirical contribution to understanding public engagement with novel foods.

**Response**: Thank you for thess important comments. Here comes responses for these points:

**Regarding Missing Supplementary Material**

*Response:* We have now included Appendix 2 (sociodemographics) in the manuscript. We have also provided the full survey instrument (exact wording, response options, and real-time feedback) in the Supporting information (S1 Appendix, English master version). The survey was fielded in German, Spanish, and Swedish using professional translation; the exact country-language versions used for fielding will be supplied as Supporting information files at resubmission to ensure full transparency for all languages.

**Regarding Technical Errors**

*Response:* We corrected the reported technical issues, including typographical and formatting errors, and we verified consistency of the grant number and the reference list (including removal of any duplicate Hellwig et al. entries). We also checked for and corrected any instances of improper capitalization at sentence starts.

**Regarding Conclusion**

*Response:* We agree the Conclusion should not only list communication recommendations. We revised the Conclusion to include a synthesizing statement of the study’s broader empirical contribution—namely, providing a cross-national baseline of domain-specific factual knowledge gaps and demonstrating that agreement with broad, positively framed statements is best interpreted as general beliefs/openness rather than measured “acceptance” or deep understanding, before presenting practical implications and research directions.

**Reviewer #1:**

This manuscript examines public knowledge and perceptions of fungi-based foods across Germany, Spain, and Sweden using a large-scale survey of 6,004 adults. The study addresses an important topic at the intersection of sustainable food systems, consumer behavior, and science communication. The cross-national design, substantial sample size, and systematic coverage of multiple knowledge domains represent clear strengths.

Comment #1:

Regarding originality and significance, the manuscript makes a useful contribution by quantifying baseline knowledge about fungi-based foods across European populations. The finding that favorable attitudes coexist with limited process literacy is genuinely interesting. However, I would encourage revisiting the claim at lines 52-53 that this topic remains unstudied. Your own prior publications have examined consumer engagement with fungi-based foods, so the contribution should be positioned more precisely in relation to this existing work.

Response: Thank you for this helpful suggestion. We agree that our original framing could be interpreted as implying that public engagement with fungi-based foods is “unstudied,” which is not accurate given our prior publications and the broader literature. We have revised the Introduction to explicitly acknowledge existing work on consumer engagement, perceptions, and acceptance-relevant attitudes (including our own), and we have positioned the present study’s contribution more precisely. Specifically, we now frame this study as complementary to prior qualitative and attitudinal research by providing the first large-scale, cross-national quantification of targeted factual knowledge gaps and misconceptions (terminology/taxonomy, cultivation timelines, sustainability and nutrition statements) across Germany, Spain, and Sweden. This refinement clarifies that the novelty lies in the cross-national prevalence estimates of specific knowledge gaps, rather than claiming the topic has not been studied. Therefore, we revised in Introduction (study contribution paragraph): revised to cite prior publications and specify the paper’s distinct contribution as cross-national quantification of specific factual knowledge gaps (rather than an “unstudied” claim); and (b) in Results & discussion / implications language: maintained alignment with the revised framing that the study quantifies knowledge gaps and belief-like agreement patterns rather than measuring acceptance.

**Comment #2:**

Concerning methodology, the survey approach is appropriate for descriptive research. However, several concerns warrant acknowledgment. The taxonomy question combines two distinct claims, namely whether fungi and mushrooms are equivalent and whether fungi are plants. A respondent with partial understanding cannot express this within the true/false format. The discussion should acknowledge that correct responses may encompass different types of understanding. The sustainability and nutrition questions show very high endorsement rates of 82-88%, suggesting possible ceiling effects. The positive framing may invite acquiescence. I recommend tempering interpretation and acknowledging that endorsement of positively framed statements may not indicate deep process literacy. Given the numerous statistically significant subgroup differences reported across demographic variables, I recommend either applying correction for multiple comparisons or cautioning readers that specific subgroup effects should be interpreted with appropriate caution.

Response: We appreciate the reviewer’s attention to the issue of multiple comparisons and agree that this required explicit treatment. Accordingly, subgroup analyses are now adjusted for multiple testing using False Discovery Rate control according to the Benjamini–Hochberg procedure. This approach is described in the revised Section 2.4 (Data analyses). Adjustments were applied within families of related tests, defined as outcomes within the same subgroup and country. The revised Supplementary Materials report both unadjusted and FDR-adjusted p-values, with statistical significance assessed on the basis of the adjusted values and clearly indicated in the tables. Furthermore, the Results section now explicitly frames subgroup analyses as exploratory in nature, and interpretation focuses on patterns that remain robust after FDR correction and that are theoretically consistent, particularly with respect to education and dietary identity, as suggested by the reviewer. The Discussion and Conclusion have also been aligned with this framing by acknowledging the hypothesis-generating character of the subgroup analyses and cautioning against over-interpretation.

We further agree that reporting effect sizes alongside tests of statistical significance substantially improves the interpretability of subgroup comparisons. In response to this comment, we have extended the analyses and revised both the manuscript and the Supplementary Materials. Effect sizes are now reported for all subgroup comparisons described in the Results. Specifically, Cramer’s V is provided for subgroup comparisons based on contingency tables, and odds ratios with 95% confidence intervals are reported for key binary outcomes, notably for correct identification of mycoprotein. Section 2.4 (Data analyses) has been revised to explicitly describe the calculation and reporting of these effect size measures. In addition, the revised Supplementary Materials now include comprehensive tables reporting effect sizes for all subgroup analyses referenced in the manuscript, allowing readers to assess both the magnitude and direction of effects. In Sections 3.1–3.3 of the Results, we now explicitly refer to effect sizes when interpreting subgroup patterns, with particular emphasis on education- and diet-related differences, and direct readers to the corresponding tables in the Supplementary Materials.

**Comment #3:**

For results and discussion, the German taxonomy findings deserve additional exploration. The 60% incorrect rate substantially exceeds rates in Spain and Sweden. Your explanation regarding generational changes in biology education would presumably apply similarly across countries. Consider whether linguistic or other national-level factors might explain this pattern. The finding that Swedish vegans and vegetarians showed lower accuracy on taxonomy appears inconsistent with your broader narrative. The post-hoc explanation is speculative and contradicts findings elsewhere showing vegans and flexitarians perform better on other questions. I suggest acknowledging this inconsistency more directly. The nutrition knowledge discussion draws on protein synthesis literature to validate respondents' endorsement, but the survey question specifically mentions protein and amino acids, so high agreement on this dimension is expected and potentially circular.

Response: Thank you for these constructive suggestions. We agree that the German taxonomy result warrants additional exploration beyond a single educational-cohort explanation, that the Swedish vegan/vegetarian pattern should be acknowledged as inconsistent with the broader narrative, and that our nutrition discussion should avoid any appearance of circular “validation” using protein/amino-acid literature. We revised Sections 3.1 and 3.5 accordingly.

**Comment #4:**

Technical corrections required include the following: line 76 contains a spelling error where recruitement should be recruitment; line 197 has a formatting artifact where fungiand should read fungi and; line 538 begins with a lowercase letter; the grant number at line 567 reads 2013-02018 but appears as 2023-02018 in submission materials; the reference list contains a duplicate entry for Hellwig et al. appearing both as Submitted and as published in 2024; and Appendix 2 describing the sociodemographic profile is referenced but does not appear in supplementary materials.

**Response**: We corrected the error in line X and it now reads ‘recruitment’; removed the formatting artifact in line X (where fungiand should read fungi and); corrected the lowercase letter in line X; the grant number in line X; the reference list; and added the referenced sociodemographic profile as Appendix 2 in the supplementary materials. Thank you very much for your attentiveness and for notifying us of these errors.

**Reviewer #2:**

The manuscript addresses a timely and increasingly visible topic: public understanding of fungi based foods across three European countries. The study benefits from a large sample (n = 6,004), clear organization, and a direct focus on terminology, taxonomy, cultivation timelines, sustainability beliefs, and nutritional perceptions. The cross-country comparison and subgroup analyses offer useful descriptive insights. Despite these strengths, several conceptual, methodological, and interpretive issues require attention to enhance the contribution of the paper.

**Comment #1:**

The conceptualization of “knowledge” and “process literacy” requires clearer grounding. The manuscript frames its aim as assessing “public literacy” and “foundational concepts” (e.g., “limited process literacy,” “knowledge of cultivation cycles,” “foundational concepts such as the difference between fungi and mushrooms and plants”). However, the operationalization relies solely on five quiz style questions that capture factual recognition rather than literacy in a theoretical sense. The manuscript should more clearly delineate whether these single items are intended to capture conceptual knowledge, semantic familiarity, or a broader literacy construct, and avoid describing them as literacy without justification.

**Response**: Thank you for this important clarification request. We agree that our original phrasing could be read as equating “literacy” with performance on five closed-ended items, which would overstate what the instrument can support. We revised the manuscript to explicitly define the measured construct as domain-specific factual knowledge and semantic familiarity related to fungi-based foods and to treat the five questions as single-item indicators (not a validated literacy scale). We also revised the terminology throughout to avoid “public literacy” and “process literacy” claims, using more precise language such as process-related factual knowledge about cultivation timelines and semantic familiarity with terminology. To make the operationalization transparent, we now state explicitly that the items cover distinct facets: taxonomy classification, familiarity with the term “mycoprotein,” process-related knowledge about cultivation timelines, and belief-like agreement with positively framed sustainability and nutrition statements. We emphasize that these measures capture recognition/endorsement rather than broader theoretical literacy (e.g., conceptual mastery or functional application). We have therefore revised:

- Introduction (conceptual framing paragraph): rewritten to define the construct as domain-specific factual knowledge/semantic familiarity and to classify what each item measures; explicitly states the items are not interpreted as a “literacy” scale.
- Materials and methods (2.1 Data collection): rewritten to describe the five items as intentionally brief, single-item indicators of specific knowledge gaps or claim-related beliefs, not a comprehensive literacy measure.
- Results/Discussion language cleanup: replaced remaining “process literacy” phrasing with “process-related factual knowledge” where applicable.

**Comment #2:**

The validity of the quiz style measures needs fuller discussion. Several items rely on terminology that may not be consistently understood by the public. For instance, the authors treat “Proteins in fungi and mushrooms” as the only correct answer to “What is mycoprotein?” while acknowledging later that terminology in markets and regulatory contexts is inconsistent (“the term is often used to refer specifically to biomass obtained when fungi are cultivated”). Similarly, the cultivation time item may confuse mushroom cultivation with filamentous fungi (“respondents might assume that filamentous fungi require similar cultivation times to mushrooms,”). These issues suggest that item responses may reflect semantic ambiguity rather than genuine misconceptions.

**Response:** Thank you for this comment. We agree that some item responses may reflect semantic ambiguity and differential familiarity with technical terminology, rather than purely “misconceptions”. We revised the manuscript to (i) explicitly describe the mycoprotein item as testing familiarity with a common usage rather than a single authoritative definition, given variability across market/regulatory contexts; and (ii) explicitly acknowledge that the cultivation-time item may be influenced by unfamiliarity with the term “filamentous fungi” and respondents’ tendency to map the question onto mushroom cultivation, such that incorrect or uncertain responses may partly reflect semantic ambiguity. To ensure transparency, we added this caution in the Methods (item interpretation paragraph), reinforced it in the Results introduction to Section 3.3, and integrated it into the Limitations section. We also emphasize that future work should use cognitive interviewing and/or alternative item designs (e.g., separate mushroom vs. filamentous fungi timelines; less technical phrasing) to improve construct validity in cross-national surveys. The revisions were made at:

- 2.1 Data collection: expanded item-level validity cautions to include cultivation timeline semantic ambiguity alongside mycoprotein variability.
- 3.3 Awareness of cultivation duration: revised the framing to state the item likely captures both process-related factual knowledge and semantic familiarity.
- Limitations: added explicit language that both mycoprotein and cultivation timeline responses may partly reflect terminology ambiguity, not only misconceptions.

**Comment #3:**

The reliance on an opt in online panel limits generalizability and requires more explicit qualification. The study uses a YouGov panel assembled through advertising, affiliate networks, and referrals. While quality checks are described, the manuscript does not discuss how nonprobability sampling, online only inclusion, or panel conditioning may shape estimates of knowledge. Given that the paper frequently refers to “the sampled populations” or “broadly shared perceptions,” the limitations of the sampling frame and potential biases among digitally engaged respondents should be explicitly addressed.

**Response:** We agree and have strengthened the sampling and generalizability qualifications. The manuscript now states clearly that the YouGov data come from a nonprobability, online-only opt-in panel, and therefore prevalence estimates should be interpreted as applying to the survey samples rather than population parameters. We explicitly note that online-only recruitment can exclude individuals with limited internet access and that digitally engaged panel members may differ systematically from the general population (e.g., education, income, media use, interest in novel foods). We also added a specific caveat regarding panel conditioning (repeated survey participation potentially influencing familiarity with survey topics/formats), and we state that post-stratification weights cannot fully remove unobserved selection biases. In addition, we reviewed wording in the Results/Discussion and replaced language implying population inference (e.g., “sampled populations”) with more accurate language (e.g., “survey samples”). The revisions are carried out at:

- Section 2.2 Panel recruitment: added explicit statements on nonprobability sampling, online-only inclusion, and panel conditioning, and clarified interpretation of prevalence estimates.
- Limitations: expanded generalizability caveats and explicitly added panel conditioning and residual selection bias.
- Results (3.3): replaced “across the sampled populations” with “across the three national survey samples.”
- Appendix 2: retained as the required sociodemographic profile to support evaluation of sample composition.

**Comment #4:**

Statistical reporting is partially incomplete, preventing assessment of substantive significance. The manuscript states that column proportion tests were computed with p < 0.05 and then presents dozens of subgroup comparisons, many of which are significant due to very large sample size. However, no effect sizes, confidence intervals, or adjustments for multiple comparisons are reported. Without indicators of magnitude, readers cannot evaluate how meaningful these differences are. Adding effect sizes and acknowledging the elevated risk of Type I errors would strengthen the analytical rigor.

**Response**: We appreciate the reviewer’s attention to the issue of multiple comparisons and agree that this required explicit treatment. Accordingly, subgroup analyses are now adjusted for multiple testing using False Discovery Rate control according to the Benjamini–Hochberg procedure. This approach is described in the revised Section 2.4 (Data analyses). Adjustments were applied within families of related tests, defined as outcomes within the same subgroup and country. The revised Supplementary Materials report both unadjusted and FDR-adjusted p-values, with statistical significance assessed on the basis of the adjusted values and clearly indicated in the tables. Furthermore, the Results section now explicitly frames subgroup analyses as exploratory in nature, and interpretation focuses on patterns that remain robust after FDR correction and that are theoretically consistent, particularly with respect to education and dietary identity, as suggested by the reviewer. The Discussion and Conclusion have also been aligned with this framing by acknowledging the hypothesis-generating character of the subgroup analyses and cautioning against over-interpretation.

We further agree that reporting effect sizes alongside tests of statistical significance substantially improves the interpretability of subgroup comparisons. In response to this comment, we have extended the analyses and revised both the manuscript and the Supplementary Materials. Effect sizes are now reported for all subgroup comparisons described in the Results. Specifically, Cramer’s V is provided for subgroup comparisons based on contingency tables, and odds ratios with 95% confidence intervals are reported for key binary outcomes, notably for correct identification of mycoprotein. Section 2.4 (Data analyses) has been revised to explicitly describe the calculation and reporting of these effect size measures. In addition, the revised Supplementary Materials now include comprehensive tables reporting effect sizes for all subgroup analyses referenced in the manuscript, allowing readers to assess both the magnitude and direction of effects. In Sections 3.1–3.3 of the Results, we now explicitly refer to effect sizes when interpreting subgroup patterns, with particular emphasis on education- and diet-related differences, and direct readers to the corresponding tables in the Supplementary Materials.

**Comment #5:**

Interpretive claims at times extend beyond what descriptive, cross-sectional data can support. Several explanations for subgroup differences are speculative. For example, lower accuracy among Swedish vegans and vegetarians is attributed to “conceptually dichotomizing foods into plant based versus animal-based categories”, and Swedish correctness on mycoprotein is linked to “a strong contemporary mushroom foraging and consumption culture”. Because the study does not measure cognitive schemas, cultural exposure, or neophobia, these interpretations should be clearly identified as hypotheses rather than empirically derived conclusions.

**Response:** We agree. The prior phrasing could be read as explanatory rather than hypothesis-generating. We have revised the Discussion to clearly separate (i) observed descriptive patterns from (ii) contextual hypotheses that cannot be tested with the present design. We now explicitly flag interpretations about cognitive schemas, cultural exposure, and related mechanisms as hypotheses for future work. Revision in manuscript are performed on Discussion text in Sections 3.2–3.3 was rewritten to use “one possible hypothesis…” language and to state that variables such as exposure/culture/schemas were not measured and therefore cannot be inferred.

**Comment #6:**

The conclusion that positive perceptions indicate “soft acceptance” is overstated based on available data. The manuscript infers that high agreement with sustainability and nutritional statements “may indicate a form of soft acceptance”. However, the survey does not measure attitudes, acceptance, willingness to try, or affective evaluations. Responding “true” to general sustainability or nutritional statements does not necessarily indicate acceptance of fungi-based foods. The authors should avoid inferring attitudinal constructs that were not measured.

**Response:** We agree. Agreement with positively framed statements should not be interpreted as acceptance. We revised the manuscript to avoid inferring attitudinal constructs (affective, cognitive, behavioral) that were not measured and to interpret these items as perceived plausibility/openness to claims, with possible acquiescence and ceiling effects. Revision in manuscript: All “soft acceptance” framing was removed; sustainability/nutrition items are now discussed as belief/claim endorsement, not acceptance.

**Comment #7:**

The discussion of nutritional parity oversimplifies a complex nutritional domain. The statement “fungi based food can offer a nutritional profile comparable to meat based products” is treated as unequivocally correct, yet the authors themselves later note that micronutrient content differs (“iron and vitamin B12 contents can be lower”). Respondents answering “false” may not lack knowledge but may consider micronutrients part of a nutritional profile. The manuscript should clarify the criterion for correctness and acknowledge that nutritional parity is conditional on the nutritional dimension considered.

**Response:** We agree and have clarified the correctness criterion and interpretation. The survey statement foregrounded protein/amino acids but also used the phrase “nutritional profile,” which can reasonably be interpreted more broadly (e.g., micronutrients). We now acknowledge that a nutritionally sophisticated respondent could answer “false” depending on their interpretation of “nutritional profile,” and we emphasize that this single item does not measure comprehensive nutrition literacy. Revision were carried out on Section 3.5 now (i) tightens language to protein/amino-acid comparability, (ii) clarifies why “true” was coded as correct under the survey’s intended focus, and (iii) explicitly notes the broader-profile interpretation as a plausible reason for “false”.

**Comment #8:**

The integration of sustainability literature at times blends respondent beliefs with scientific evidence. In Section 3.4, the manuscript shifts from reporting survey results (“85 percent…answered the correct answer ‘true’”) to summarizing life cycle assessments and global modeling. This structure may blur the line between empirical perceptions and scientific evidence. Separating these elements more clearly would prevent the impression that high public agreement validates sustainability claims.

**Response:** We agree and revised the structure to keep a clean separation between (1) what respondents endorsed and (2) the scientific evidence base. We now label evidence summaries explicitly as context (not validation of survey agreement). Revision were performed on Section 3.4 was edited so the survey findings are presented first, followed by a clearly marked “scientific evidence context” paragraph, with an explicit statement that the evidence is not used to validate the survey responses.

**Comment #9:**

The extensive subgroup analyses would benefit from stronger theoretical anchoring or parsimony. The manuscript reports numerous significant subgroup differences across education, age, income, diet, region, and ethnicity. However, the discussion frequently attributes these differences to psychological constructs that were not measured (e.g., food neophobia, masculinity linked norms). A more cautious interpretation or a more focused theoretical framework would help avoid overinterpreting descriptive patterns.

**Response:** We agree. We revised the interpretation of subgroup differences to be more parsimonious and cautious, focusing on robust gradients (especially education and diet identity) and explicitly treating other subgroup findings as descriptive and hypothesis-generating. We removed/avoided attributing differences to unmeasured constructs (e.g., masculinity norms, neophobia). Revision were carried out on Discussion paragraphs interpreting subgroup differences were rewritten to remove strong psychological attributions and to emphasize that mediators were not measured.

**Comment #10:**

The limitations section should more fully account for methodological constraints. Missing elements include the use of single item measures without validated psychometric properties, the potential for translation differences across languages, the inability to assess whether respondents interpreted items consistently across countries, the lack of behavioral outcomes, and the large number of statistical tests increasing false positive risk. A fuller acknowledgement would improve transparency.

**Response:** We agree and expanded the Limitations to address: single-item measures without psychometric validation; potential translation/wording differences across languages and lack of measurement invariance testing; inability to verify consistent item interpretation across countries; cross-sectional design and absence of behavioral outcomes; and the risk of false positives due to many subgroup tests (noting our effect size reporting and FDR adjustment, but still urging caution). For the revision, Limitations expanded accordingly.

**Comment #11:**

The manuscript would benefit from integrating recent evidence on how education shapes sustainability related beliefs and behaviors, which directly parallels several of the manuscript’s findings on knowledge gradients. One useful study (<https://www.mdpi.com/2071-1050/13/23/13036>) shows that higher educational attainment predicts stronger pro environmental attitudes and more consistent sustainable behavioral intentions, demonstrating that education functions not only as a sociodemographic descriptor but as a structural determinant of sustainability engagement. Incorporating this evidence would strengthen the interpretation of the manuscript’s repeated observation that respondents with tertiary education were more likely to answer correctly across multiple domains (“higher knowledge was observed among tertiary educated groups”). It would also support the manuscript’s argument that educational targeting is necessary to address knowledge gaps. In addition, recent research on health literacy and nutrition related judgments (<https://www.mdpi.com/2304-8158/14/3/378>) suggests that health literacy can mediate the pathway between education and informed behavioral outcomes. This is relevant because the manuscript interprets misunderstandings of terminology, cultivation processes, and nutritional claims as barriers to informed decision making (“limited process literacy…underscores the need for clearer communication”). Drawing on evidence that health literacy transmits part of education’s effect on sustainable or health aligned choices would help clarify why educational gradients emerge so consistently in the current study, and why communication strategies should be designed to improve not only factual knowledge but also evaluative competencies that support sustainable dietary behaviors.
In my view, the manuscript offers valuable descriptive insights into public understanding of fungi based foods but requires substantial refinement in conceptual framing, measurement discussion, statistical transparency, and interpretive caution. I recommend a major revision.

**Response:** Thank you. This is helpful and aligns with our consistent education gradients. We incorporated recent evidence showing that education predicts sustainability-related dietary attitudes/intentions and that health literacy can mediate pathways between education and nutrition-related behaviors, strengthening our interpretation of education as a structural determinant rather than merely a descriptor. Therefore, we added these citations in the Discussion where we interpret the repeated education gradients and where we motivate education-targeted communication strategies.
